# Surgical treatment of giant cell tumors of the sacrum and spine combined with pre-operative transarterial embolization

**DOI:** 10.3892/ol.2013.1329

**Published:** 2013-05-08

**Authors:** MING ZHOU, HUILIN YANG, KANGWU CHEN, GENLIN WANG, JIAN LU, YIMING JI, CHUNSHEN WU, CHAO CHEN, HAI HU

**Affiliations:** Department of Orthopedic Surgery, The First Affiliated Hospital of Soochow University, Suzhou, Jiangsu 215006, P.R. China

**Keywords:** giant cell tumor, blood loss, spine, sacrum, embolization

## Abstract

The pre-operative embolization of hypervascular spinal tumors is often performed to decrease intraoperative blood loss and facilitate tumor resection; however, few studies have been published on its effectiveness in giant cell tumors (GCT) of the sacrum and spine. The purpose of the present study was to investigate the value of surgical excision with pre-operative transarterial embolization for GCTs of the sacrum and spine, and to evaluate the follow-up outcomes. A retrospective study was performed on 28 patients with GCTs of the sacrum and spine, who underwent surgical treatment combined with pre-operative transarterial embolization between June 1995 and August 2011. The intraoperative blood loss, transfusion, duration of surgery, treatment, local recurrence, complications, follow-up status and functional outcome were reviewed. The average follow-up period was 86.3 months (range, 12–193 months). All the patients were treated with intralesional resection without any intraoperative shock or fatalities. The average intraoperative level of blood loss was 1,528.6 ml (range, 400–5,800 ml), the average transfusion volume was 1,514.3 ml (range, 400–6,000 ml) and the average duration of surgery was 225.4 min (range, 120–470 min). In total, eight (28.6%) patients developed recurrence and two patients succumbed. A total of eight (28.6%) patients experienced complications and 24 (85.7%) retained normal neurological function. Pre-operative embolization significantly decreases intraoperative blood loss and facilitates the maximal removal of the tumor. Pre-operative embolization followed by intralesional resection is able to achieve satisfactory local control and clinical outcomes. It is an effective technique for excising GCTs of the sacrum and spine.

## Introduction

Although giant cell tumors (GCT) of the bones are relatively common primary bone tumors, it is rare for a GCT to occur in the sacrum and spine (1–3). The symptoms are not specific, and the tumors are difficult to diagnose at the early stage. The tumors may attain a large size and neurological deficits may be experienced secondary to compression of the spinal cord or nerve roots (4–6). Large spinal GCTs are difficult to manage. Although the optimal treatment of GCTs in the sacrum and spine remains controversial, surgery is the main treatment; complete excision is recommended for the treatment of spinal GCTs.

Compared with the normal spine, GCT is a hypervascular lesion (1,7). Massive blood loss during surgery is a severe complication (3,8,9), which may be life-threatening and make the surgery impossible to complete (10). Therefore, pre-operative embolization of spinal tumors is recommended to reduce intraoperative bleeding and make an unresectable tumor resectable. Several studies have reported results for the pre-operative embolization of hypervascular spinal tumors and clearly showed that the technique is safe and effective at reducing intraoperative blood loss (11–13). However, only a few studies have examined the effect of pre-operative embolization for GCTs of the spine and the majority are case reports (14).

The purpose of the present retrospective study was to investigate the value of surgical excision with pre-operative transarterial embolization for GCTs of the sacrum and spine, and evaluate the follow-up outcomes.

## Materials and methods

### Patient data

A total of 28 consecutive patients (16 females and 12 males) with GCTs of the sacrum and spine, who underwent surgical excision combined with pre-operative transarterial embolization between June 1995 and August 2011, were retrospectively reviewed. The average age at the first diagnosis was 29.6 years (range, 11–58 years). All medical charts were reviewed, including the clinical records, operative notes and radiological and histological findings. All 28 patients were verified to have GCT by histological examination following surgery. Of the 28 cases, 13 were located in the mobile spine, with 8 thoracic and 5 lumbar GCTs, and 15 cases were located in the sacrum. The majority of patients presented with pain or a neurological deficit at the site of tumor involvement, such as paresthesia, weakness and bowel and/or bladder dysfunction. The duration of the pre-operative symptoms ranged between 0.5 and 40 months (median, 4 months). Patients were followed up via clinical examination and using imaging studies at the outpatient clinic every three months for two years and then every six months thereafter. The intraoperative level of blood loss, transfusion, duration of surgery, treatment, complications, local recurrence, follow-up status and functional outcome were reviewed. Patient demographics are shown in Table I. This study was approved by the ethics committee of Soochow University. Written informed consent was obtained from all patients.

### Pre-operative arterial embolization

All patients underwent pre-operative angiography and embolization using the reformed Seldinger methods following femoral artery insertion under local anesthesia. The tumor-feeding vessels and the size and location of the tumor, as well as the association between the blood supply arteries and surrounding tissues, were determined by digital subtraction angiography. Particular attention was paid to the visualization of the radiculomedullary branches to the anterior spinal artery (artery of Adamkiewicz). If the artery of Adamkiewicz was visualized in the pre-embolization digital view, embolization of this segmental vessel was not performed.

In the mobile spine, devascularization of the segmental vessels was attempted bilaterally at the level of the lesion, as well as at the cephalic and caudal levels, while in the sacrum, the arteries embolized included the bilateral internal iliac, middle sacral and bilateral L4 transverse arteries.

**Figure 1.**
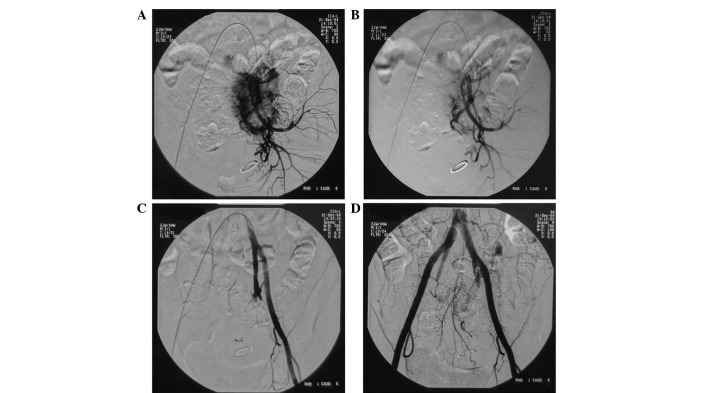
Digital subtraction angiography, anteroposterior view. (A) Insertion of a catheter into the internal iliac artery to take an angiogram; (B) small intratumoral arteries embolized with gelfoam particles; (C) embolization of the stem of the tumor-feeding artery using a gelfoam strip; and (D) complete embolization of all tumor-feeding arteries.

**Figure 2.**
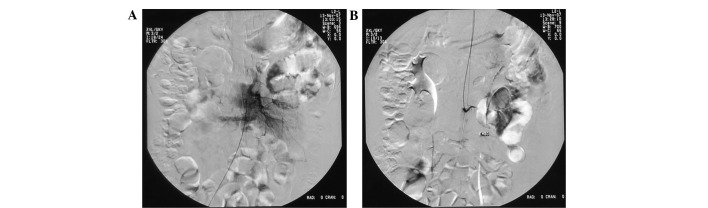
Angiogram prior to and following selective arterial embolization. (A) Preembolization angiogram showing hypervascular tumor blush at L3. (B) Postembolization angiogram showing no appreciable residual tumor blush, but complete devascularization of the giant cell tumor.

Gelfoam particles were used to embolize the small intratumoral arteries, and the stems of the tumor-feeding arteries were embolized with a gelfoam strip. The angiograph was performed again to ensure that all tumor-supplying vessels were embolized. (Figs. 1 and 2).

### Operative technique

Surgery was performed within one to two days after the arterial embolization. For the mobile spine, the surgical approach was decided based on the position of the tumor. If the stabilization of the spine was disrupted following the removal the tumor, instrumentation was required. For the GCTs of the sacrum, a single posterior approach was used for 14 patients, while a one-stage anterior and posterior combined approach was used for one patient who presented with a larger tumor. The mechanical stability was often insufficient if the S1 and total sacrum or substantial portions of the two iliac wings were excised; consequently, a spinal instrumentation system was required to support the spine. All the tumor excisions were defined as intralesional. Subsequent to exposure, the tumor perimeter was packed with gauze to prevent spillage of the tumor tissue during curettage. The nerve roots were protected and preserved whenever possible. If the nerve roots or spinal dura mater were contaminated with the tumor cell during surgery, the membrane was dissected carefully. The surgical field was covered thoroughly with 95% ethyl alcohol gauze, then cleaned with warm normal saline. Sterilized distilled water was used to lyse the residual microscopic tumor debris. Routinely, effective suction drainage was placed post-operatively to promote primary healing.

### Statistical analysis

Data were analyzed with SAS version 8.1 (SAS Institute, Cary, NC, USA). For comparisons of the quantitative data of the two groups, the independent samples t-test was used; continuous data are expressed in terms of the mean and standard deviation. P<0.05 was considered to indicate a statistically significant difference.

## Results

No symptomatic complications were observed to be associated with embolization, and all the tumor masses were removed completely without any intraoperative shock or fatalities. The average intraoperative level of blood loss was 1,528.6 ml (range, 400–5,800 ml), the average transfusion volume was 1,514.3 ml (range, 400–6,000 ml) and the average duration of surgery was 225.4 min (range, 120–470 min). The sites of the tumors and the lengths of time between embolization and tumor resection were compared in terms of average intraoperative level of blood loss, average transfusion volume and average duration of surgery (Table II).

All the patients were treated with intralesional surgical resection combined with pre-operative transarterial embolization. A total of 14 patients underwent reconstruction. Six patients were treated post-operatively with adjuvant radiation therapy and no radiation myelopathy or sarcomatous transformation was observed in the study. Of the 28 patients, eight (28.6%) experienced complications perioperatively or during the follow-up. Six (21.4%) patients had wound complications; three experienced skin necrosis, two had wound infections and one patient had a sinus tract infection. Five of these patients were healed following dressing changes, debridement or systemic antibiotics, although the remaining patient still suffered from wound exudation at the final follow-up, which required dressing changes every day. One patient experienced cerebrospinal fluid leakage that was treated by a conservative method. The dural tears were repaired with a 4-0 or 5-0 silk suture and then covered with a gelatin sponge. The patient was positioned with their head down, in the Trendelenburg position. One thoracic patient developed kyphosis, which was also treated conservatively as there was no pain and the patient was able to tolerate the condition. No patients experienced deep-vein thrombosis, pulmonary embolism or hardware failure requiring surgical revision.

**Figure 3.**
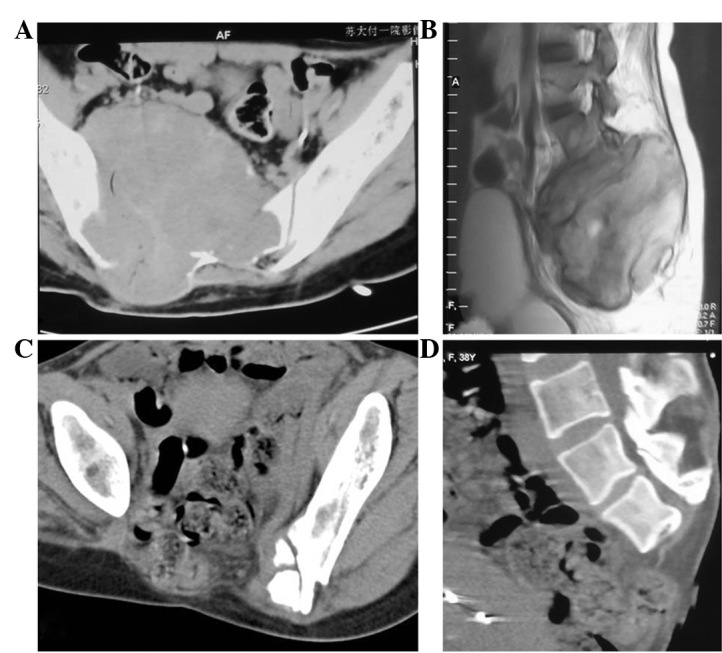
A 29-year-old female with a large giant cell tumor affecting the sacrum. (A) Axial CT scan showing an osteolytic lesion in the sacrum. (B) Sagittal T2-weighted MRI revealing a large soft tissue extension but no involvement of the surrounding tissue. (C) Axial and (D) sagittal CT scan of a 105 months post-surgical excision with pre-operative embolization showing no tumor recurrence. CT, computed tomography; MRI, magnetic resonance imaging.

**Figure 4.**
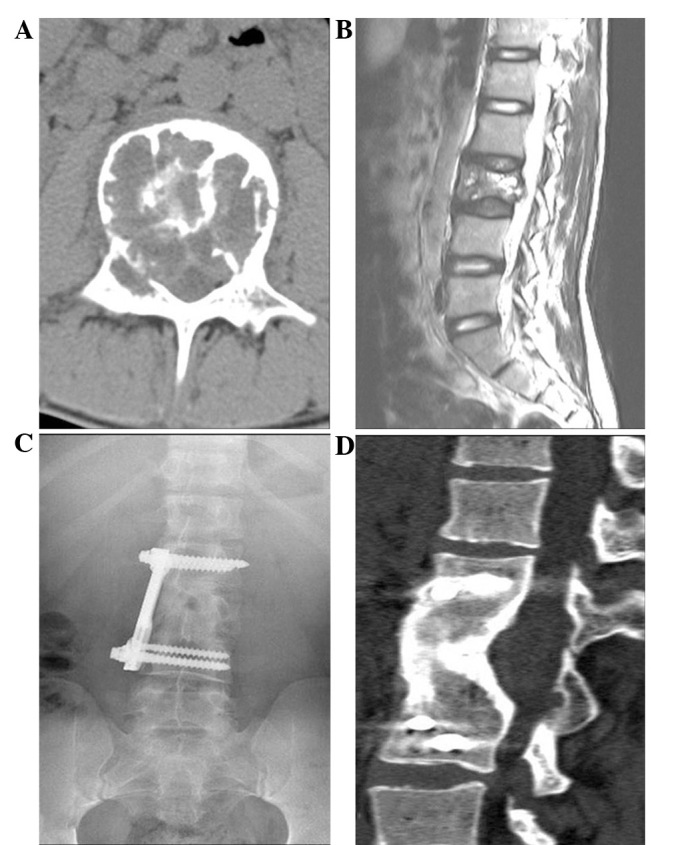
A 16-year-old male with a giant cell tumor affecting the L3 vertebra. (A) Axial CT scan of the L3 vertebra showing an osteolytic lesion in the vertebral body; (B) Sagittal T2-weighted MRI revealing invasion of the mass through the posterior wall of the vertebral body into the spinal canal; (C) Anteroposterior radiograph and (D) sagittal CT scan at 73 months follow-up, showing no recurrence. CT, computed tomography; MRI, magnetic resonance imaging.

All the cases were followed up for an average of 86.3 months (range, 12–193 months). A total of eight patients developed recurrence (28.6%) and the average time was 35.6 months (range, 5–79 months). Of the eight patients, seven received surgical revision with no repeated recurrence observed. The remaining patient received no treatment and remained alive with the disease. At the final follow-up, 25 patients showed no evidence of disease, one patient was alive with the disease and two patients had succumbed. No patients had metastases in the lungs. In total, 11 sacral GCT patients (Fig. 3) and all the spinal GCT patients (13 cases; Fig. 4) had normal neurological function, whereas the function of the sphincter muscles was impaired in four sacral GCT patients. Two patients were able to walk with an assistive device and the other 26 patients were able to ambulate without any support.

## Discussion

GCTs are hypervascular lesions (1,7) that rarely occur in the spine (1–3). Due to the complicated anatomical structure and hypervascularity of the tumors, massive blood loss often occurs during the surgical treatment procedures. Turcotte *et al* (3) reported that the average level of intraoperative blood loss was 7,500 ml, while Ozaki *et al* (8) reported that the level of intraoperative blood loss ranged between 2,400 and 6,700 ml (median, 5,250 ml). Takeda *et al* (9) reported the level of intraoperative blood loss of two patients to be 4,921 ml and 20,000 ml, respectively. Bleeding during spinal GCT surgery is a severe complication, which may be life-threatening and make it impossible to complete the surgical procedure (10). Therefore, pre-operative embolization of the spinal tumors is recommended to reduce intraoperative bleeding. Several studies have reported the results of pre-operative embolization for hypervascular spinal tumors and have clearly shown that the technique is safe and effective at reducing intraoperative blood loss (11–13). In a study on spinal metastases from renal cell carcinoma (15), the median intraoperative blood loss was recorded as 1,500 ml (range 300–8,000 ml). Wilson *et al* (16) reported that the mean estimated level of intraoperative blood loss of the primary spine tumors was 1,562 ml. Similarly in the present study, the estimated mean level of blood loss was 1,528.6 ml.

In the present study, a gelatin sponge was used as the embolic agent, which is a temporary vascular occlusive agent that is degraded by proteolytic enzymatic pathways and resorbed within seven to 21 days after embolization (17). The majority of authors suggest that surgery should be performed within 24 h to prevent pre-operative recanalization or tumor revascularization via collaterals (12). We agree that subsequent surgery should be performed within 24 h, but the present study observed that the level of blood loss was usually not large if embolization occurred within two days. The average level of blood loss of patients who underwent surgery within one day post-embolization was 1,626.1 ml and the average level of blood loss of patients who underwent surgery within two days was 1,080 ml; no significant difference was observed (P>0.05). In the present study, gelfoam particles were used to embolize the small intratumoral arteries and then gelfoam strips were used to embolize the stem of the tumor-feeding artery. With this method, all tumor-feeding arteries may be embolized completely and the risk of hemorrhage from anastomoses of the lateral branches may be decreased.

Pre-operative transarterial embolization for tumors of the spine is a relatively safe procedure, but it does carry certain risks. The most catastrophic complication is spinal cord ischemia associated with the embolization of unrecognized radiculomedullary arteries. Finstein *et al* (18) reported a case of post-embolization paralysis and paresthesia in a patient with a thoracolumbar GCT. Although, this complication is extremely rare, careful analysis of the pre-embolization angiograms is essential to identify and protect the radiculomedullary and spinal arteries. Angiography should be performed prior to embolization to define the vascular anatomy. The presence of a radiculomedullary artery supplying the spinal cord and a dangerous intersegmental anastomosis should not be missed.

Although histologically benign, GCTs are locally aggressive and the local recurrence rate is significantly higher in the spine compared with the extremities. Sanjay *et al* (4) reported a recurrence rate of 42% from 24 patients with GCTs of the spine at the Mayo Clinic. Although, the optimal treatment of GCTs in the sacrum and spine remains controversial, complete excision is recommended. Theoretically, en bloc resection with either a marginal or wide resection margin is able to decrease the recurrence rate, although the duration of surgery, level of blood loss and perioperative complication rates of this procedure are high (19,20). In the review of Cloyd *et al* (21), the duration of surgery averaged 12.1 h and lasted as long as 42 h, while the mean blood loss was 3.7 liters, with one patient losing as much as 37 liters. Certain authors (8,9,22) have recommended conservative surgery (curettage or intralesional excision), which has a lower morbidity and less neurological deficits. These are accompanied by certain other advantages, including the preservation of the stability of the spine and pelvis, the speed and ease of the surgical procedure and the potential for reduced blood loss. In the present study, all the patients underwent pre-operative transarterial embolization, which reduced the intraoperative blood loss and allowed the surgical margin of the tumor to be identified clearly. The recurrence rate of the present study was 28.6%, which is lower than or equal to that reported in the literature (4,23).

The complications observed included kyphosis, cerebrospinal fluid leakage and wound complications. Wound complications occurred in six sacral cases and are the most frequent complications following a posterior approach in the sacrum, requiring long and intensive treatment (6,24). We suggest that two effective suction drainage tubes should be placed to aid in the prevention of hematomas developing in the large dead spaces.

The functional outcomes of the present study were satisfactory. In the sacrum, 13 of the 15 patients remained active with a full range of motion in their lower extremities, while the remaining two patients were able to walk with sticks. Of the 15 patients with sacrum GCT, four patients experienced bowel or bladder dysfunction. When bilateral S2 nerve roots and the unilateral S3 nerve root were preserved, 8 of 10 patients had normal bowel and bladder function. Bilateral S2 nerve roots were preserved in 3 patients and all of them had normal bowel and bladder function. In the remaining 2 patients, only the unilateral S2 nerve roots were preserved and all of them had bowel and bladder dysfunction. This is similar to the study by Todd *et al* (24), which demonstrated that the preservation of at least the unilateral S3 nerve root is extremely important for patients in order to sustain normal bowel and bladder function. In the mobile spine, all 13 patients had no residual neurological deficits at final follow-up and were able to perform normal daily activities. However, Martin *et al* (23) reported that seven out of 13 spinal GCT patients had either chronic pain or residual neurological deficits. These may be attributed to pre-operative transarterial embolization, which is used to reduce intraoperative hemorrhaging and provide a clear surgical field and adequate curettage. We suggest that if the nerve roots or spinal dura mater is contaminated with the tumor cell during surgery, the membrane should be dissected carefully. Following the removal of the tumor, the surgical field was covered thoroughly with 95% ethyl alcohol gauze, then cleaned with warm normal saline. Sterilized distilled water was used to lyse the residual microscopic tumor debris.

Compared with techniques used in the historical literature, pre-operative embolization significantly decreases the level of intraoperative blood loss, makes the surgical field clear and facilitates the maximal removal of the tumor. Pre-operative embolization followed by intralesional resection is able to achieve satisfactory local control and clinical outcomes. It is an effective technique for excising GCTs of the sacrum and spine. However, further comprehensive studies are required and a control group would be necessary to strengthen the results.

## Figures and Tables

**Table I.** Patient demographics.

Demographic	Data
Gender, n	
Male	12
Female	16
Age (years), n	
<20	3
20–40	19
>40	6
Mean	29.6
Site, n	
Sacrum	15
Lumbar spine	5
Thoracic spine	8
Pre-operative neurological function, n	
Normal	11
Partial loss	17
Complete loss	0
Surgical approach, n	
A	8
P	18
A+P	2
Reconstruction, n	
Yes	14
No	14
Nerve root sacrificed, n	
Yes	13
No	15
Local recurrence, n	
Yes	8
No	20
Neurological function at six months, n	
Normal	24
Partial loss	4
Complete loss	0
Last status, n	
NED	25
STU	2
AWD	1

A, anterior; P, posterior; NED, no evidence of disease; AWD, alive with disease; STU, succumbed to unrelated disease.

**Table II.** Blood loss, transfusion and duration of surgery.

Variable	Patient no.	Blood loss (ml)			Transfusion (ml)			Surgery duration (min)		
		Mean	SD	P-value (t-test)	Mean	SD	P-value (t-test)	Mean	SD	P-value (t-test)
Site				0.611			0.641			0.266
Sacrum	15	1640.0	1455.4		1620.0	1472.7		204.7	104.2	
Mobile Spine	13	1400.0	899.1		1392.3	986.1		249.2	102.7	
Time between embolization and surgery (days)				0.372			0.302			0.140
1	23	1626.1	1257.8		1630.4	1308.9		245.0	110.0	
2	5	1080.0	965.4		980.0	861.4		167.0	60.2	
